# Local amplification of highly pathogenic avian influenza H5N8 viruses in wild birds in the Netherlands, 2016 to 2017

**DOI:** 10.2807/1560-7917.ES.2018.23.4.17-00449

**Published:** 2018-01-25

**Authors:** Marjolein J. Poen, Theo M. Bestebroer, Oanh Vuong, Rachel D. Scheuer, Henk P. van der Jeugd, Erik Kleyheeg, Dirk Eggink, Pascal Lexmond, Judith M.A. van den Brand, Lineke Begeman, Stefan van der Vliet, Gerhard J.D.M. Müskens, Frank A. Majoor, Marion P.G. Koopmans, Thijs Kuiken, Ron A.M. Fouchier

**Affiliations:** 1 Erasmus MC, Department of Viroscience, Rotterdam, the Netherlands; 2 Netherlands Institute of Ecology (NIOO-KNAW), Department of Animal Ecology, Wageningen, the Netherlands; 3 Vogeltrekstation – Dutch Centre for Avian Migration and Demography (NIOO-KNAW), Wageningen, the Netherlands; 4 Academic Medical Center Amsterdam, Laboratory of Experimental Virology, Amsterdam, the Netherlands; 5 Alterra, Center for Ecosystem Studies, Wageningen University, Wageningen, the Netherlands; 6 Sovon, Dutch Centre for Field Ornithology, Nijmegen, the Netherlands

**Keywords:** avian influenza, emerging or re-emerging diseases, influenza virus, outbreaks, surveillance, Viral infections

## Abstract

Highly pathogenic avian influenza (HPAI) viruses of subtype H5N8 were re-introduced into the Netherlands by late 2016, after detections in south-east Asia and Russia. This second H5N8 wave resulted in a large number of outbreaks in poultry farms and the deaths of large numbers of wild birds in multiple European countries. **Methods**: Here we report on the detection of HPAI H5N8 virus in 57 wild birds of 12 species sampled during active (32/5,167) and passive (25/36) surveillance activities, i.e. in healthy and dead animals respectively, in the Netherlands between 8 November 2016 and 31 March 2017. Moreover, we further investigate the experimental approach of wild bird serology as a contributing tool in HPAI outbreak investigations. **Results**: In contrast to the first H5N8 wave, local virus amplification with associated wild bird mortality has occurred in the Netherlands in 2016/17, with evidence for occasional gene exchange with low pathogenic avian influenza (LPAI) viruses. **Discussion**: These apparent differences between outbreaks and the continuing detections of HPAI viruses in Europe are a cause of concern. With the current circulation of zoonotic HPAI and LPAI virus strains in Asia, increased understanding of the drivers responsible for the global spread of Asian poultry viruses via wild birds is needed.

## Introduction

Highly pathogenic avian influenza (HPAI) viruses of the H5 subtype, originating from the A/Goose/Guangdong/1/1996 (GsGd) lineage, have been circulating continuously in poultry in south-east Asia since 1997 and have also been detected frequently in wild birds [1]. In 2014, a new HPAI H5N8 virus of this GsGd lineage of clade 2.3.4.4 emerged globally. This first intercontinental wave of HPAI H5N8 started with virus detections in south-east Asia from early 2014 onwards in both poultry and wild birds [2-4]. By the end of 2014, this HPAI H5N8 virus simultaneously spread to Europe and North America through long distance migratory birds [5]. In North America, the virus reassorted with local low pathogenic avian influenza (LPAI) viruses causing a massive number of outbreaks and associated economical loss [6]. In Europe, this first wave caused a relative limited number of outbreaks in poultry holdings, and was detected in some wild birds between November 2014 and February 2015 [7,8]. During the spring and summer of 2015, occasional detections of HPAI H5N8 were reported in south-east Asia [9]. To assess the risk of virus re-introduction by wintering birds arriving in Europe by the autumn of 2015, intensified active surveillance (i.e. surveillance in living birds) was performed in the Netherlands from September to December 2015. This surveillance provided virological and serological evidence that the HPAI H5N8 virus had disappeared from the European (wintering) wild bird population with no virus detections in any of the tested birds and a decreased seroprevalence of HPAI H5 clade 2.3.4.4-specific antibodies, suggesting no massive viral replication in the 2015 breeding season [10].

However, in June 2016, the detection of HPAI H5N8 in wild birds of multiple species on their breeding grounds was reported around Uvs-Nuur Lake in Russia [11]. In contrast to the 2014 emerging strains, which belong to group A (A/broiler duck/Korea/Buan2/2014-like), this virus belonged to group B (A/breeder duck/Korea/Gochang1/2014-like) viruses [4,11]. These group B viruses had been detected previously in China and South Korea in 2014, but had not been reported since [3,12]. From mid-October 2016 onwards, group B lineage HPAI H5N8 viruses were detected in both India [13] and in European countries. Unlike the 2014/15 group A viruses, group B viruses caused local die-offs of wild birds in many countries, often resulting in wild bird deaths preceding those in poultry [14-16]. The introduction of these group B HPAI H5N8 viruses in the Netherlands was marked by a die-off of tufted ducks (*Aythya fuligula*) and great crested grebes (*Podiceps cristatus*) in the Gouwzee (52°27’09”N, 5°04’07”E), a large fresh water lake, on 8 November 2016 [14,17]. Most of the internal genes of this ‘second wave’ HPAI H5N8 virus were derived from Eurasian LPAI viruses via reassortment, after their original detection in China and South Korea in 2014 and Russia in May 2016 [11,15]. Occasional reassortment of the neuraminidase (NA) gene also led to a few detections of clade 2.3.4.4 HPAI H5N5 and H5N6 viruses [18].

Wild migratory birds were shown to be the most probable vectors for the first global spread of HPAI H5N8 in 2014 that coincided with the timing and flyways of the autumn migration, based on a recent worldwide phylogenetic study of HPAI H5N8 viruses [5,19]. These avian influenza viruses constitute a constant animal and human health threat, where the risk in part is determined by the (evolving) genomic constitution of the circulating viruses. It is therefore of crucial importance to actively monitor influenza viruses and their evolution in wild bird populations, to monitor trends and diversity of circulating viruses, and to assess the risk of spread for strains that are unusual in their genetic make-up and/or spread for animal and human health. In this study we have performed intense active surveillance in wild birds in the Netherlands in response to the HPAI H5N8 introduction in Europe in late 2016. We performed both virological and serological studies to attempt to identify wild bird species that might contribute to the spread and maintenance of this virus.

## Methods

### Ethical statement

The capture of free-living birds was approved by the Dutch Ministry of Economic Affairs based on the Flora and Fauna Act (permit number FF/75A/2009/067, FF/75A/2014/054 and licence number 951 to Vogeltrekstation NIOO-KNAW). Handling and sampling of free-living birds was approved by the Animal Experiment Committee of the Erasmus Medical Center (permit number 122–11–31). Free-living birds were released into the wild after sampling and all efforts were made to minimise animal suffering throughout the procedures.

### Study population

A continuous active surveillance programme of resident and migrating wild birds for avian influenza viruses is in place in the Netherlands. The ongoing surveillance efforts were intensified in response to the first detection of HPAI H5N8 virus in the Netherlands in 2016 between 13 November and 31 December 2016, the period during which mortality among wild birds and outbreaks in poultry holdings were occurring in the country, and from 8 February until 19 February 2017, when die-offs of wild birds and outbreaks in poultry had ceased. On 1 March and 23 October 2016, as well as approximately during the first period of intensified surveillance (13 November to 21 December 2016), and on 8 February 2017, blood samples were obtained in addition to samples for virus detection.

### Sample collection

Live wild birds were captured using duck decoys, cannon nets, leg nooses, swan hooks, or manually. Birds were sampled routinely for virus detection using oropharyngeal and cloacal swabs as described elsewhere [10]. In addition, fresh faecal samples were collected from Eurasian wigeons (*Anas penelope*) for virus detection. Fresh faecal samples were collected by trained ornithologists able to distinguish species-specific droppings from locations where large homogeneous groups of Eurasian wigeons were foraging in the field. Blood samples were collected for serum antibody detection as described previously [10]. In addition to active surveillance, oropharyngeal and/or cloacal swabs of a limited number of freshly dead wild birds were opportunistically collected for virus detection (i.e. passive surveillance).

### Virus detection, isolation and characterisation

Samples for virus detection were analysed for the presence of HPAI H5(N8) virus using matrix- and H5-specific real-time reverse-transcription PCR (RRT-PCR) assays, followed by haemagglutinin (HA) and NA gene sequencing as previously described [7]. Samples testing positive in matrix and H5 specific RRT-PCR were inoculated in Madin–Darby canine kidney (MDCK) cells. Samples were characterised as HPAI H5 virus by detection of a multi-basic cleavage site upon Sanger sequencing of the HA gene.

### Virus sequencing and phylogeny

Full length HA and NA sequences of all virus isolates and full genome sequences for a subset of these were obtained by Sanger sequencing. All sequences were deposited in a public database (http://www.gisaid.com). Primer sequences are available upon request.

For HA and NA phylogeny, sequences obtained in this study were supplemented with publicly available sequences of HPAI H5 viruses of clade 2.3.4.4 detected globally between 2014 and 2017. These additional sequences were obtained from the Global Initiative on Sharing Avian Influenza Data database (http://www.gisaid.com) on 20 May 2017 (Table 1).

**Table 1:** Acknowledgements of authors, and originating and submitting laboratories providing the sequences used for phylogenetic analysis

Isolate ID	Isolates name	Collection date	Originating laboratory	Submitting Laboratory	Authors
EPI_ISL_257700	A/Mute_Swan/Czech_Republic/1813–17/2017	5 Feb 2017	Not listed	State Veterinary Institute Prague	Nagy, A.
EPI_ISL_250885	A/Mute_Swan/Czech_Republic/54–17_2/2017	2 Jan 2017	Not listed	State Veterinary Institute Prague	Nagy, A.
EPI_ISL_248663	A/Mallard/Czech_Republic/722–17_2/2017	15 Jan 2017	Not listed	State Veterinary Institute Prague	Nagy, A.
EPI_ISL_247713	A/Tufted_Duck/Denmark/17740–1/2016	8 Nov 2016	Technical University of Denmark	Animal and Plant Health Agency (APHA)	Hjulsager CK, Krog JS, Larsen LE, Kvisgaard LK, Essen S
EPI_ISL_238197	A/Mute_Swan/Croatia/78/2016	12 Nov 2016	Not listed	Croatian Veretinary Institute	Not listed
EPI_ISL_238196	A/Mute_Swan/Croatia/70/2016	30 Oct 2016	Not listed	Croatian Veretinary Institute	Not listed
EPI_ISL_237945	A/Tufted_Duck/Germany/AR8459-L01988/2016	8 Nov 2016	Not listed	Friedrich-Loeffler-Institut	Not listed
EPI_ISL_237944	A/Tufted_Duck/Germany/AR8444-L01987/2016	7 Nov 2016	Not listed	Friedrich-Loeffler-Institut	Not listed
EPI_ISL_237732	A/Tufted_Duck/Germany-SH/R8446/2016	7 Nov 2016	Not listed	Friedrich-Loeffler-Institut	Not listed
EPI_ISL_238039	A/Chicken/Germany-SH/R8758/2016	11 Nov 2016	Not listed	Friedrich-Loeffler-Institut	Not listed
EPI_ISL_237921	A/Wild_Duck/Poland/82A/2016	2 Nov 2016	Not listed	National Veterinary Research Institut Poland	Świętoń E, Śmietanka K
EPI_ISL_175535	A/MuteSwan/Sweden/SVA150313KU0141/SZ543/2015	5 Mar 2015	National Veterinary Institute, Sweden	National Veterinary Institute, Sweden	Zohari S, Ullman K, Olofsson A
EPI_ISL_238896	A/Chicken/Sweden/SVA161122KU0453/SZ0209321/2016	21 Nov 2016	National Veterinary Institute, Sweden	National Veterinary Institute, Sweden	Not listed
EPI_ISL_231685	A/Black-headed_Gull/Tyva/41/2016	25 May 2016	State Research Center of Virology and Biotechnology (VECTOR)	WHO National Influenza Centre Russian Federation	Fadeev A, Komissarov A, Egorova A, Sintsova K, Musaeva T, Susloparov I, Marchenko V, Ryzhikov A
EPI_ISL_231684	A/Wild_Duck/Tyva/35/2016	25 May 2016	State Research Center of Virology and Biotechnology (VECTOR)	WHO National Influenza Centre Russian Federation	Fadeev A, Komissarov A, Egorova A, Sintsova K, Musaeva T, Susloparov I, Marchenko V, Ryzhikov A
EPI_ISL_224580	A/Great_Crested_Grebe/Tyva/341/2016	25 May 2016	Research Institute of Experimental and Clinical Medicine	Research Institute of Experimental and Clinical Medicine	Sharshov K, Kurskaya O, Sobolev I, Alekseev A, Alikina T, Kabilov M, Shestopalov A
EPI_ISL_234057	A/grey_heron/Uvs-Nuur_Lake/20/2016	25 May 2016	Research Institute of Experimental and Clinical Medicine	Research Institute of Experimental and Clinical Medicine	Sharshov K, Kurskaya O, Sobolev I, Alekseev A, Shestopalov A
EPI_ISL_234058	A/common_tern/Uvs-Nuur_Lake/26/2016	25 May 2016	Research Institute of Experimental and Clinical Medicine	Research Institute of Experimental and Clinical Medicine	Sharshov K, Kurskaya O, Sobolev I, Alekseev A, Alikina T, Kabilov M, Shestopalov A
EPI_ISL_269601	A/Eurasian_Herring_Gull/Netherlands/2/2016	20 Dec 2016	Erasmus Medical Center	Erasmus Medical Center	Poen,MJ, Van Der Jeugd,HP, Vuong,O, Scheuer,RD, Fouchier,RAM et al.
EPI_ISL_268916	A/Caspian _Gull/Netherlands/1/2016	20 Dec 2016	Erasmus Medical Center	Erasmus Medical Center	Poen,MJ, Van Der Jeugd,HP, Vuong,O, Scheuer,RD, Fouchier,RAM et al.
EPI_ISL_255910	A/Mew Gull/Netherlands/1/2016	23 Nov 2016	Erasmus Medical Center	Erasmus Medical Center	Poen,MJ, Van Der Jeugd,HP, Vuong,O, Scheuer,RD, Fouchier,RAM et al.
EPI_ISL_269602	A/Lesser_Black-backed_Gull/Netherlands/1/2016	20 Dec 2016	Erasmus Medical Center	Erasmus Medical Center	Poen,MJ, Van Der Jeugd,HP, Vuong,O, Scheuer,RD, Fouchier,RAM et al.
EPI_ISL_269697	A/Great_Black-backed_Gull/Netherlands/2/2016	23 Nov 2016	Erasmus Medical Center	Erasmus Medical Center	Poen,MJ, Van Der Jeugd,HP, Vuong,O, Scheuer,RD, Fouchier,RAM et al.
EPI_ISL_269599	A/Great_Black-backed_Gull/Netherlands/4/2016	14 Dec 2016	Erasmus Medical Center	Erasmus Medical Center	Poen,MJ, Van Der Jeugd,HP, Vuong,O, Scheuer,RD, Fouchier,RAM et al.
EPI_ISL_269598	A/Great_Black-backed_Gull/Netherlands/3/2016	23 Nov 2016	Erasmus Medical Center	Erasmus Medical Center	Poen,MJ, Van Der Jeugd,HP, Vuong,O, Scheuer,RD, Fouchier,RAM et al.
EPI_ISL_269597	A/Great_Black-backed_Gull/Netherlands/1/2016	23 Nov 2016	Erasmus Medical Center	Erasmus Medical Center	Poen,MJ, Van Der Jeugd,HP, Vuong,O, Scheuer,RD, Fouchier,RAM et al.
EPI_ISL_255892	A/Great Black-backed Gull/Netherlands/2/2016	23 Nov 2016	Erasmus Medical Center	Erasmus Medical Center	Poen,MJ, Van Der Jeugd,HP, Vuong,O, Scheuer,RD, Fouchier,RAM et al.
EPI_ISL_269600	A/Great_Crested_Grebe/Netherlands/2/2016	21 Dec 2016	Erasmus Medical Center	Erasmus Medical Center	Poen,MJ, Van Der Jeugd,HP, Vuong,O, Scheuer,RD, Fouchier,RAM et al.
EPI_ISL_269696	A/Eurasian_Wigeon/Netherlands/23/2016	05 Dec 2016	Erasmus Medical Center	Erasmus Medical Center	Poen,MJ, Van Der Jeugd,HP, Vuong,O, Scheuer,RD, Fouchier,RAM et al.
EPI_ISL_269694	A/Eurasian_Wigeon/Netherlands/1/2016	04 Dec 2016	Erasmus Medical Center	Erasmus Medical Center	Poen,MJ,Müskens,G.J.D.M, Vuong,O, Scheuer,RD, Fouchier,RAM et al.
EPI_ISL_269596	A/Eurasian_Wigeon/Netherlands/13/2016	14 Dec 2016	Erasmus Medical Center	Erasmus Medical Center	Poen,MJ, Van Der Jeugd,HP, Vuong,O, Scheuer,RD, Fouchier,RAM et al.
EPI_ISL_269595	A/Eurasian_Wigeon/Netherlands/12/2016	14 Dec 2016	Erasmus Medical Center	Erasmus Medical Center	Poen,MJ, Van Der Jeugd,HP, Vuong,O, Scheuer,RD, Fouchier,RAM et al.
EPI_ISL_269594	A/Eurasian_Wigeon/Netherlands/22/2016	14 Dec 2016	Erasmus Medical Center	Erasmus Medical Center	Poen,MJ, Van Der Jeugd,HP, Vuong,O, Scheuer,RD, Fouchier,RAM et al.
EPI_ISL_269593	A/Eurasian_Wigeon/Netherlands/11/2016	13 Dec 2016	Erasmus Medical Center	Erasmus Medical Center	Poen,MJ, Van Der Jeugd,HP, Vuong,O, Scheuer,RD, Fouchier,RAM et al.
EPI_ISL_269592	A/Eurasian_Wigeon/Netherlands/8/2016	09 Dec 2016	Erasmus Medical Center	Erasmus Medical Center	Poen,MJ, Van Der Jeugd,HP, Vuong,O, Scheuer,RD, Fouchier,RAM et al.
EPI_ISL_269591	A/Eurasian_Wigeon/Netherlands/6/2016	09 Dec 2016	Erasmus Medical Center	Erasmus Medical Center	Poen,MJ, Van Der Jeugd,HP, Vuong,O, Scheuer,RD, Fouchier,RAM et al.
EPI_ISL_268937	A/Eurasian_Wigeon/Netherlands/10/2016	08 Dec 2016	Erasmus Medical Center	Erasmus Medical Center	Poen,MJ, Van Der Jeugd,HP, Vuong,O, Scheuer,RD, Fouchier,RAM et al.
EPI_ISL_255914	A/Eurasian Wigeon/Netherlands/9/2016	04 Dec 2016	Erasmus Medical Center	Erasmus Medical Center	Poen,MJ, Van Der Jeugd,HP, Vuong,O, Scheuer,RD, Fouchier,RAM et al.
EPI_ISL_255912	A/Eurasian Wigeon/Netherlands/4/2016	09 Dec 2016	Erasmus Medical Center	Erasmus Medical Center	Poen,MJ, Van Der Jeugd,HP, Vuong,O, Scheuer,RD, Fouchier,RAM et al.
EPI_ISL_269703	A/Eurasian_Wigeon/Netherlands/25/2016	05 Dec 2016	Erasmus Medical Center	Erasmus Medical Center	Poen,MJ, Van Der Jeugd,HP, Vuong,O, Scheuer,RD, Fouchier,RAM et al.
EPI_ISL_269695	A/Eurasian_Wigeon/Netherlands/21/2016	05 Dec 2016	Erasmus Medical Center	Erasmus Medical Center	Poen,MJ, Van Der Jeugd,HP, Vuong,O, Scheuer,RD, Fouchier,RAM et al.
EPI_ISL_269692	A/Mallard/Netherlands/3/2017	11 Jan 2017	Erasmus Medical Center	Erasmus Medical Center	Poen,MJ, Van Der Jeugd,HP, Vuong,O, Scheuer,RD, Fouchier,RAM et al.
EPI_ISL_269604	A/Mallard/Netherlands/1/2017	07 Jan 2017	Erasmus Medical Center	Erasmus Medical Center	Poen,MJ, Van Der Jeugd,HP, Vuong,O, Scheuer,RD, Fouchier,RAM et al.
EPI_ISL_269603	A/Mallard/Netherlands/51/2016	20 Dec 2016	Erasmus Medical Center	Erasmus Medical Center	Poen,MJ, Van Der Jeugd,HP, Vuong,O, Scheuer,RD, Fouchier,RAM et al.
EPI_ISL_255913	A/Mallard/Netherlands/2/2017	07 Jan 2017	Erasmus Medical Center	Erasmus Medical Center	Poen,MJ, Van Der Jeugd,HP, Vuong,O, Scheuer,RD, Fouchier,RAM et al.
EPI_ISL_268927	A/Common_Buzzard/Netherlands/1/2016	07 Dec 2016	Erasmus Medical Center	Erasmus Medical Center	Poen,MJ, Van Der Jeugd,HP, Vuong,O, Scheuer,RD, Fouchier,RAM et al.
EPI_ISL_255891	A/Tufted Duck/Netherlands/1/2016	25 Nov 2016	Erasmus Medical Center	Erasmus Medical Center	Poen,MJ, Van Der Jeugd,HP, Vuong,O, Scheuer,RD, Fouchier,RAM et al.
EPI_ISL_268866	A/Back-headed_Gull/Netherlands/9/2016	20 Dec 2016	Erasmus Medical Center	Erasmus Medical Center	Poen,MJ, Van Der Jeugd,HP, Vuong,O, Scheuer,RD, Fouchier,RAM et al.
EPI_ISL_268800	A/Black-headed_Gull/Netherlands/17/2016	20 Dec 2016	Erasmus Medical Center	Erasmus Medical Center	Poen,MJ, Van Der Jeugd,HP, Vuong,O, Scheuer,RD, Fouchier,RAM et al.
EPI_ISL_268799	A/Back-headed_Gull/Netherlands/8/2016	20 Dec 2016	Erasmus Medical Center	Erasmus Medical Center	Poen,MJ, Van Der Jeugd,HP, Vuong,O, Scheuer,RD, Fouchier,RAM et al.
EPI_ISL_268929	A/Common_Eider/Netherlands/2/2016	20 Dec 2016	Erasmus Medical Center	Erasmus Medical Center	Poen,MJ, Van Der Jeugd,HP, Vuong,O, Scheuer,RD, Fouchier,RAM et al.
EPI_ISL_269693	A/Common_Pochard/Netherlands/1/2016	25 Nov 2016	Erasmus Medical Center	Erasmus Medical Center	Poen,MJ, Van Der Jeugd,HP, Vuong,O, Scheuer,RD, Fouchier,RAM et al.

Maximum likelihood (ML) phylogenetic trees were constructed based on the HA (1,637 nt: position 49–1,685) and NA (1,227 nt: position 64–1,291) genes. ML trees were generated using PhyML version 3.1 using the general time-reversible (GTR) model, performing subtree pruning and regrafting (SPR) searches [20]. The reliability of the phylogenetic grouping was assessed with 250 bootstrap replicates. Trees were visualised using Figtree version 1.4.3 (http://tree.bio.ed.ac.uk/software/figtree). ML trees of HA and NA were used in Dendroscope version 3.5.9 (http://dendroscope.org/) [21] to display a tanglegram between the HA and NA midpoint rooted phylogenies. The twines were colour-coded according to wave and location.

### Antibody detection

Serum samples were initially screened for the presence of clade 2.3.4.4 H5(N8)-specific (A/Chicken/Netherlands/EMC-3/2014 and A/Great Black-backed Gull/Netherlands/3/2016) and LPAI H5(N2)-specific (A/Mallard/Netherlands/3/1999) antibodies in a haemagglutination inhibition (HI) assay according to standard procedures [10,22]. Due to the generally high non-specific haemagglutination induced by wild bird sera in previous HI assays [10], all sera were pre-treated with 10% turkey red blood cells for 1 hour at 4 °C before analysis. Negative controls, based on incubation of serum without virus, were used to measure non-specific haemagglutination of each serum sample. Serum samples from experimentally inoculated ferrets [23] were used as positive controls.

Serum samples that tested positive for either LPAI H5N2 or HPAI H5 clade 2.3.4.4-specific antibodies were further tested in an HI assay against HPAI viruses of the H5 clades 1 (A/Viet Nam/1194/2004), 2.1 (A/Indonesia/5/2005), 2.2 (A/Turkey/Turkey/1/2005), and 2.3.4 (A/Anhui/1/2005), and retested against the 2016 clade 2.3.4.4 virus (A/Great Black-backed gull/Netherlands/3/2016). The viruses used, except the 2016 HPAI H5N8 virus and the LPAI H5N2 virus, were recombinant viruses based on an A/PR/8/34 virus backbone, containing the HA (without the multi-basic cleavage site) and NA of the representative H5 strains to enable this study within biosafety level 2 laboratories. Assays with the wild type 2016 HPAI H5N8 virus were performed simultaneously in biosafety level 3 conditions.

Subsequently, samples were tested in a virus neutralisation (VN) assay as described previously [10], using titrated virus stocks of the same LPAI H5N2 and HPAI H5 clade 1, 2.1, 2.2, 2.3.4, and 2016 2.3.4.4 representatives.

Sera were categorised as being either LPAI or HPAI biased or ambiguous, where a bias is defined as a cut-off of > 1 log_2_ differences in titre in HI assays [24].

## Results

### Study population

**Figure 1:**
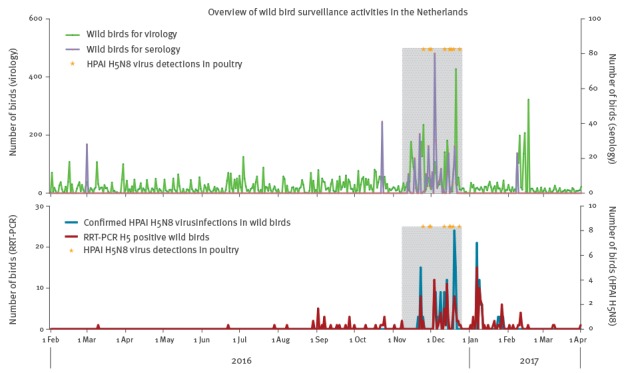
Overview of wild bird surveillance activities in the Netherlands between 1 February 2016 and 31 March 2017, with intensified surveillance from 13 November–31 December 2016 and 8–19 February 2017

Here we report the data of 5,167 wild birds that were tested for the presence of avian influenza viruses between 8 November 2016 and 31 March 2017 in response to the re-introduction of HPAI H5N8 viruses in the Dutch wild bird population on 8 November 2016 [17]. In addition, we report on all data obtained in our routine active surveillance activities before the first evidence of re-introduction of HPAI H5N8 virus into the Netherlands, 1 February until 7 November 2016 (n = 5,523) (Table 2, Figure 1). All birds were caught alive and did not show clinical signs of disease. Also, samples were obtained from 36 birds belonging to 17 species that were sampled post mortem (Table 2).

**Table 2:** Wild bird species sampled for virus detection in the Netherlands before and during the second wave of highly pathogenic avian influenza H5N8 virus in Europe and results of virological assays, February 2016–March 2017 (n = 10,726)

Order	Family	Species	1 February 2016–7 November 2016				8 November 2016–31 March 2017							
			Alive without clinical signs				Alive without clinical signs				Found dead			
			Number of birds Sampled	Number of birds AIV positive	Number of birds H5 positive	Pathotype	Number of birds sampled	Number of birds AIV positive	Number of birds H5 positive	Pathotype	Number of birds sampled	Number of birds AIV positive	Number of birds H5 positive	Pathotype
*Anseriformes*	Ducks	Common eider *(Somateria mollissima)*	0	0	0	NA	0	0	0	NA	1	1	1	1x HPAI
		Common pochard *(Aythya ferina)*	0	0	0	NA	0	0	0	NA	1	1	1	n.i.
		Common shelduck *(Tadorna tadorna)*	2	0	0	NA	0	0	0	NA	0	0	0	NA
		Domestic duck *(Anas platyrhynchos domesticus)*	0	0	0	NA	1	0	0	NA	0	0	0	NA
		Egyptian goose *(Alopochen aegyptiaca)*	30	0	0	NA	17	0	0	NA	0	0	0	NA
		Eurasian teal *(Anas crecca)*	46	21	0	NA	42	5	1	1x LPAI	0	0	0	NA
		Eurasian wigeon *(Anas penelope)*	639	339	10	6x n.i., 4x LPAI	2,634	118	37	14x HPAI, 23x n.i.	7	7	7	7x HPAI
		Gadwall *(Anas strepera)*	131	65	0	NA	11	1	1	1x n.i.	1	1	0	NA
		Garganey *(Anas querquedula)*	2	2	0	NA	0			NA	0	0	0	NA
		Greater scaup *(Aythya marila)*	1	0	0	NA	2	1	0	NA	0	0	0	NA
		Mallard *(Anas platyrhynchos)*	3,169	555	20	4x n.i., 16x LPAI	1,824	338	78	17x HPAI, 5x LPAI, 56x n.i.	3	3	2	2x HPAI
		Northern pintail *(Anas acuta)*	12	9	0	NA	6	0	0	NA	0	0	0	NA
		Northern shoveler *(Anas clypeata)*	14	4	0	NA	3	0	0	NA	0	0	0	NA
		Tufted duck *(Aythya fuligula)*	2	1	0	NA	2	0	0	NA	1	1	1	1x HPAI
	Geese	Barnacle goose *(Branta leucopsis)*	589	0	0	NA	0			NA	0	0	0	NA
		Bean goose *(Anser fabalis)*	0	0	0	NA	1	0	0	NA	0	0	0	NA
		Canada goose *(Branta canadensis)*	23	0	0	NA	3	0	0	NA	0	0	0	NA
		Great white-fronted goose *(Anser albifrons)*	27	0	0	NA	40	0	0	NA	0	0	0	NA
		Greylag goose *(Anser anser)*	310	3	1	1x LPAI	0			NA	0	0	0	NA
	Swans	Bewick's swan *(Cygnus columbianus bewickii)*	0	0	0	NA	92	3	0	NA	0	0	0	NA
		Mute swan *(Cygnus olor)*	0	0	0	NA	36	0	0	NA	0	0	0	NA
		Whooper swan *(Cygnus cygnus)*	0	0	0	NA	3	0	0	NA	0	0	0	NA
*Charadriiformes*	Gulls	Black-headed gull *(Chroicocephalus ridibundus)*	432	59	0	NA	287	2	0	NA	4	4	3	3x HPAI
		Caspian gull *(Larus cachinnans)*	0	0	0	NA	1	0	0	NA	1	1	0	NA
		Eurasian herring gull *(Larus argentatus)*	24	0	0	NA	20	1	0	NA	2	2	2	1x HPAI, 1x n.i.
		Great black-backed gull *(Larus marinus)*	0	0	0	NA	0	0	0	NA	8	8	5	5x HPAI
		Lesser black-backed gull *(Larus fuscus)*	66	0	0	NA	0	0	0	NA	1	1	1	1x HPAI
		Mew gull *(Larus canus)*	2	0	0	NA	13	0	0	NA	1	1	1	1x HPAI
		Yellow-legged gull *(Larus michahellis)*	0	0	0	NA	0	0	0	NA	1	0	0	NA
*Gruiformes*	Coots	Common coot *(Fulica atra)*	2	0	0	NA	100	0	0	NA	0	0	0	NA
		Common moorhen *(Gallinula chloropus)*	0	0	0	NA	4	0	0	NA	0	0	0	NA
		Water rail *(Rallus aquaticus)*	0	0	0	NA	20	0	0	NA	0	0	0	NA
*Pelicaniformes*	Ardeidae	Grey heron *(Ardea cinerea)*	0	0	0	NA	3	0	0	NA	0	0	0	NA
*Podicipediformes*	Grebes	Great crested grebe *(Podiceps cristatus)*	0	0	0	NA	0	0	0	NA	1	1	1	1x HPAI
*Suliformes*	Cormorants	Great cormorant *(Phalacrocorax carbo)*	0	0	0	NA	0	0	0	NA	1	0	0	NA
*Passeriformes*	Corvidae	Eurasian magpie *(Pica pica)*	0	0	0	NA	1	0	0	NA	0	0	0	NA
*Falconiformes*	Falcons	Peregrine falcon *(Falco peregrinus)*	0	0	0	NA	0	0	0	NA	1	1	1	1x HPAI
*Accipitriformes*	Accipitridae	Common buzzard *(Buteo buteo)*	0	0	0	NA	1	1	1	1x HPAI	1	1	1	1x HPAI
Total	NA	NA	5,523	1,058	31	NA	5,167	470	118	32x HPAI	36	34	27	25x HPAI

AIV: avian influenza virus; HPAI: highly pathogenic avian influenza; LPAI: low pathogenic avian influenza; NA: not applicable; n.i.: not identifiable because of low virus load.

RRT-PCR: real-time reverse-transcription PCR.

Displayed over time are the number of birds tested for virus presence (green line), the number of birds positive for H5 in the RRT-PCR (dark red line) with the number of confirmed HPAI H5N8 cases (blue line), and the number of birds included for serology (purple line). In addition, the period with the largest wild-bird die-offs (light grey box) and the HPAI H5N8 detections in commercial poultry in the Netherlands (orange stars) are displayed.

For antibody detection, serum samples from 459 birds of various species were analysed (Table 3). The majority of these samples were obtained between 13 November and 21 December 2016 (n = 367, 18 species) and on 8 February 2017 (n = 23 mallards *(Anas platyrhynchos*)). In addition we included blood samples from Eurasian wigeons obtained on 1 March (n = 28) and 23 October 2016 (n = 41) that were not analysed previously (Table 3).

**Table 3:** Wild bird species sampled in the Netherlands for antibody detection in response to the second wave of highly pathogenic avian influenza (HPAI) H5N8 virus in Europe, 2016/2017 (n = 459) and those showing HPAI H5 clade 2.3.4.4-specific antibodies (n = 20) based on repeated haemagglutination inhibition assays (HI), March 2016–February 2017

Order	Family	Species	Number of individuals					
			1 March 2016		23 October 2016		13 November 2016–8 February 2017	
			Tested	H5 clade 2.3.4.4- specific antibodies	Tested	H5 clade 2.3.4.4- specific antibodies	Tested	H5 clade 2.3.4.4- specific antibodies
*Anseriformes*	Ducks	Egyptian goose *(Alopochen aegyptiaca)*	0	0	0	0	10	0
		Eurasian teal *(Anas crecca)*	0	0	0	0	22	0
		Eurasian wigeon *(Anas penelope)*	28	2	41	1	63	2
		Gadwall *(Anas strepera)*	0	0	0	0	5	0
		Mallard *(Anas platyrhynchos)*	0	0	0	0	72	11
		Northern pintail *(Anas acuta)*	0	0	0	0	6	0
		Tufted duck *(Aythya fuligula)*	0	0	0	0	1	0
	Swans	Bewick's swan *(Cygnus columbianus bewickii)*	0	0	0	0	20	0
		Mute swan *(Cygnus olor)*	0	0	0	0	24	3
		Whooper swan *(Cygnus cygnus)*	0	0	0	0	3	0
*Charadriiformes*	Gulls	Black-headed gull *(Chroicocephalus ridibundus)*	0	0	0	0	88	1
		Caspian gull *(Larus cachinnans)*	0	0	0	0	1	0
		Eurasian herring gull *(Larus argentatus)*	0	0	0	0	15	0
		Mew gull *(Larus canus)*	0	0	0	0	7	0
*Gruiformes*	Rails	Common coot *(Fulica atra)*	0	0	0	0	35	0
		Water rail *(Rallus aquaticus)*	0	0	0	0	16	0
*Passeriformes*	Corvidae	Eurasian magpie *(Pica pica)*	0	0	0	0	1	0
*Pelicaniformes*	Ardeidae	Grey heron *(Ardea cinerea)*	0	0	0	0	1	0
Total	NA	NA	28	2	41	1	390	17

NA: not applicable.

### Virus detection, isolation and characterisation

There was no evidence for the presence of HPAI H5(N8) virus in any of the birds (n = 5,523) sampled during routine active surveillance between 1 February and 7 November 2016. In the subsequent period (between 8 November 2016 and 31 March 2017), samples from 145 birds (2.8%) tested positive for the presence of H5 HA by RRT-PCR. The presence of HPAI H5 clade 2.3.4.4 virus was confirmed in samples of 57 birds (Table 2). Of these, 23 birds (17 mallards, 5 Eurasian wigeons and one common buzzard (*Buteo buteo*)) were caught and sampled alive without clinical signs, and from nine birds (Eurasian wigeons) fresh droppings were tested positive. (Table 4). In total, viruses were isolated in MDCK cells from 48 samples from 33 birds. All cultured viruses belonged to the HPAI H5N8 subtype. The last detection of HPAI clade 2.3.4.4 virus in living birds was in mallards on 28 January 2017. Since then, no additional HPAI clade 2.3.4.4 H5 viruses have been detected in this study.

**Table 4:** Overview of wild birds or wild birds’ droppings, which were sampled in the Netherlands during active surveillance, and positive for highly pathogenic avian influenza H5N8 virus, 23 November 2016–28 January 2017 (n = 32 birds)

Species	Location	Status	Date	Number of animals
Eurasian wigeon	Echtenerburg	Dropping	23 November 2016	1
Eurasian wigeon	Warder	Live without clinical signs	04 December 2016	3
Common buzzard	Hippolytushoef	Live without clinical signs	07 December 2016	1
Eurasian wigeon	Oud Alblas	Live without clinical signs	08 December 2016	1
Eurasian wigeon	Oud Alblas	Live without clinical signs	12 December 2016	1
Eurasian wigeon	Nijkerk	Dropping	12 December 2016	3
Eurasian wigeon	Nijkerk	Dropping	14 December 2016	2
Eurasian wigeon	Nijkerk	Dropping	21 December 2016	3
Mallard	Oud Alblas	Live without clinical signs	07 January 2017	6
Mallard	Oud Alblas	Live without clinical signs	09 January 2017	4
Mallard	Oud Alblas	Live without clinical signs	10 January 2017	2
Mallard	Oud Alblas	Live without clinical signs	11 January 2017	2
Mallard	Oud Alblas	Live without clinical signs	24 January 2017	1
Mallard	Oud Alblas	Live without clinical signs	25 January 2017	1
Mallard	Oud Alblas	Live without clinical signs	28 January 2017	1

### Virus sequencing and phylogenetic analysis

Full length HA and NA sequences of all 48 isolates were obtained by Sanger sequencing. Analysis of these 48 samples showed no differences between sequences obtained from cloacal and oropharyngeal swabs from the same bird, so only one sequence per bird was included in further analyses.

**Figure 2:**
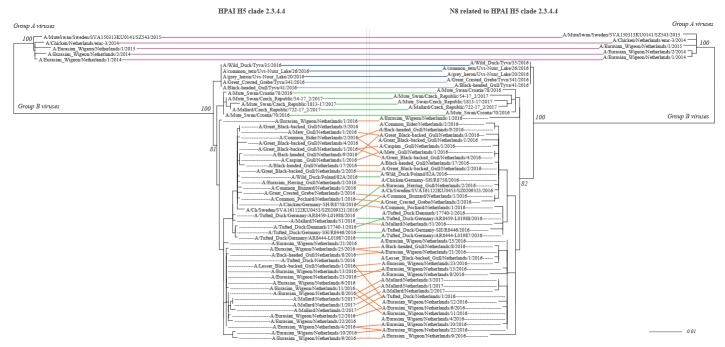
Tanglegram of highly pathogenic avian influenza H5 clade 2.3.4.4 virus (left) and the accompanying N8 genes (right) based on 250 bootstraps

In accordance with previous reports [11,15,25], our phylogenetic analysis (Figure 2) shows a clear distinction for both HA and NA between the 2014/15 group A HPAI H5N8 viruses and the 2016/17 group B viruses. Also, the Russian viruses from May 2016 were distinguishable from the ones that entered eastern European countries (Croatia and Czech Republic) and subsequently more western European counties like Germany and the Netherlands for both HA and NA. The subclade consisting only of Dutch duck and gull viruses might indicate more local virus evolution within the Netherlands. In support, the viruses detected in live mallards in early 2017 appear as offspring from Eurasian wigeon viruses that were detected 3 weeks earlier and were highly similar to other Dutch viruses that caused mortality in other bird species (Figure 2). However, the number of sequences from other outbreaks in Europe at present is too limited to draw solid conclusions.

Only bootstrap values above 70% are shown. The 2014/15 group A H5 viruses with their N8 genes (pink) are phylogenetically distinct from the 2016/17 group B viruses first detected in Russia (Tyva and Uvs-Nuur Lake) in May–June 2016 (blue) and later in other European countries (green) and the Netherlands (orange).

Full genome sequences were obtained for six isolates by Sanger sequencing. The six isolates shared 99.1–99.7% nt sequence identity across all of the eight genes in the genome. Basic local alignment search tool (BLAST, https://www.ncbi.nlm.nih.gov/blast/) search results for earlier detected viruses showed the highest identity (97.8–99.0%) with the HPAI H5N8 group B viruses for HA, NA and non-structural protein (NS). The remaining five gene segments (polymerases PB2, PB1 and PA, nucleoprotein (NP) and matrix protein (MP)) showed the highest identity with Eurasian LPAI viruses (Table 5). New reassortment events were observed for the PA and NP genes since the original detection of the HPAI H5N8 virus in Russia in May 2016.

**Table 5:** Search results for sequences^a^ with high similarity to the eight genes found in the full genomes of six highly pathogenic avian influenza H5N8 virus isolates from the Netherlands, 2016/17

Gene	BLAST result	Identity	Classification
PB2	[6/6] A/duck/Bangladesh/26920/2015(H3N6)	>98,7%	LPAI
PB1	[5/6] A/chicken/Hunan/S1267/2010(H4N6)	>97,7%	LPAI
	[1/6] A/duck/Mongolia/179/2015(H3N8)	97,5%	LPAI
PA	[6/6] A/duck/Mongolia/129/2015(H3N3)	>98,0%	LPAI
HA	[6/6] A/duck/Eastern China/S1109/2014(H5N8)	>98,7%	HPAI H5 clade 2.3.4.4
NP	[6/6] A/Mallard/Netherlands/15/2011(H6N8)	>99,2%	LPAI
NA	[6/6] A/duck/Eastern China/S1109/2014(H5N8)	>98,6%	HPAI H5 clade 2.3.4.4
MP	[6/6] A/duck/Mongolia/179/2015(H3N8)	>98,1%	LPAI
NS	[3/6] A/duck/Eastern China/S1109/2014(H5N8)	>98.8%	HPAI H5 clade 2.3.4.4
	[3/6] A/goose/Yangzhou/0420/2014(H5N8)	>97.9%	HPAI H5 clade 2.3.4.4

BLAST: basic local alignment search tool; HA: haemagglutinin; MP: matrix protein; NA: neuraminidase; NP: nucleoprotein; NS: non-structural protein; PA or PB: polymerases (PA, PB2, PB1).

^a^ Searches were carried out using the National Center for Biotechnology Information (NCBI) nucleotide-BLAST.

### Influenza A virus H5-specific antibody detection in wild birds

Seroreactivity of 459 wild bird sera was determined for different influenza H5 viruses. In a total of 29 sera, antibody titres directed to LPAI H5(N2) (A/Mallard/Netherlands/3/1999) or 2016 HPAI H5 clade 2.3.4.4 (A/Great Black-backed Gull/Netherlands/3/2016) or to both of these viruses were detected. There was good correspondence for high reacting sera (HI titre ≥ 40) between the HI antibody titres generated with the 2014 HPAI H5 clade 2.3.4.4 virus and the 2016 virus, suggesting that there has been limited antigenic drift of HPAI H5N8 viruses since 2014. In sera with lower HI titres, there was a strong bias to only react with the 2016 H5 clade 2.3.4.4 virus. When the HI assay for H5 clade 2.3.4.4-specific antibody positive sera was repeated, all but three titres were reproduced. Of the 10 sera showing antibody titres to both LPAI H5 and HPAI H5 clade 2.3.4.4 virus, one was LPAI-biased, two were HPAI-biased and seven showed ambiguous titres. Overall, 4.2% (18/431) of the sera obtained from October 2016 showed evidence of the presence of HPAI clade 2.3.4.4 H5-specific antibodies based on HI assays in duplo (Table 6) which was confirmed by VN assays in 14/18 samples from October 2016 onwards and 1/2 from 1 March 2016. 

**Table 6:** Details of the results on low pathogenic (LPAI) H5- and highly pathogenic avian influenza (HPAI) H5 clade 2.3.4.4-specific antibody positive sera using haemagglutination inhibition assays and the resulting HPAI/LPAI bias, Netherlands, 2016/17 (n = 29)

Sample ID	Species	Collection date	LPAI	HPAI H5 clade 2.3.4.4	Bias
C320-297	Black-headed gull	29 November 2016	<10	20	HPAI
320-312	Black-headed gull	05 December 2016	<10	20^a^	HPAI
309-9	Eurasian wigeon	01 March 2016	<10	20	HPAI
309-13	Eurasian wigeon	01 March 2016	<10	10	Ambiguous
309-23	Eurasian wigeon	01 March 2016	<10	20^a^	HPAI
C309-51	Eurasian wigeon	23 October 2016	40	40	Ambiguous
318-57	Eurasian wigeon	04 December 2016	<10	10^a^	Ambiguous
318-63	Eurasian wigeon	04 December 2016	40	640	HPAI
318-70	Eurasian wigeon	04 December 2016	20	<10	LPAI
318-75	Eurasian wigeon	04 December 2016	10	80	HPAI
320-55	Mallard	09 December 2016	320	40	LPAI
314-1813	Mallard	08 February 2017	<10	20	HPAI
314-1815	Mallard	08 February 2017	30	20	Ambiguous
314-1817	Mallard	08 February 2017	40	20	Ambiguous
314-1819	Mallard	08 February 2017	<10	40	HPAI
314-1820	Mallard	08 February 2017	30	<10	LPAI
314-1821	Mallard	08 February 2017	<10	10	Ambiguous
314-1823	Mallard	08 February 2017	40	<10	LPAI
314-1824	Mallard	08 February 2017	<10	120	HPAI
314-1825	Mallard	08 February 2017	40	20	Ambiguous
314-1826	Mallard	08 February 2017	40	40	Ambiguous
314-1827	Mallard	08 February 2017	10	<10	Ambiguous
314-1832	Mallard	08 February 2017	10	20	Ambiguous
314-1835	Mallard	08 February 2017	30	20	Ambiguous
320-295	Mute swan	05 December 2016	<10	30	HPAI
320-641	Mute swan	07 December 2016	<10	30	HPAI
320-699	Mute swan	14 December 2016	30	<10	LPAI
320-729	Mute swan	15 December 2016	<10	20	HPAI
320-355	Whooper Swan	20 December 2016	80	<10	LPAI

HPAI H5 clade 2.3.4.4: highly pathogenic avian influenza A/Great-black backed gull/Netherlands/3/2016 (H5N8); LPAI: low pathogenic avian influenza A/Mallard/Netherlands/3/1999 (H5N2).

^a^ Titre could not be confirmed in a second haemagglutination inhibition HI assay.

The overall HPAI H5 antibody incidence between October and December 2016 was 2.0% (8/408). However, in mallards sampled on 8 February 2017 this was 43.5% (10/23) compared with 2.0% (1/49) in mallards sampled between October and December 2016. Comparing the 2016/17 winter with the same seasons in previous years, indicated that mallards and black-headed gulls (*Chroicocephalus ridibundus)* first tested positive for HPAI H5 clade 2.3.4.4-specific antibodies in the 2016/17 winter. In contrast, for Eurasian wigeons, common coots (*Fulica atra)* and mute swans (*Cygnus olor*) the detected incidence appeared to be lower in 2016/17 compared to the 2014/15 winter (Table 7). Taking into account all the bird species considered by the surveillance over the different winters, a preliminary incidence of HPAI H5 clade 2.3.4.4.-specific antibodies can be calculated as 0% before 2014, rising to 4.6% during the first outbreak of HPAI H5N8 virus, decreasing to 3.5% in the 2015/16 winter and rising to 4.2% in the 2016/17 winter (Table 7).

**Table:** **Table 7.** Overview of highly pathogenic avian influenza H5 clade 2.3.4.4-specific antibody incidence in the Netherlands based on haemagglutination inhibition assays starting from the first wave of this virus in 2014/2015 up to February 2017

Species	2014/15^a^		2015/16^ b^		2016/17^c^	
	Positive/total	Percentage	Positive/total	Percentage	Positive/total	Percentage
Eurasian wigeon	12/78	15.4%	5/73	6.8%	3/104	2.9%
Lesser white-fronted goose	1/3	33.3%	0	U	0	U
Mute swan	29/88	33.0%	5/24	20.8%	3/24	12.5%
Common coot	1/84	1.2%	1/22	4.5%	0/35	0%
Black-headed gull	0/262	0.0%	0/31	U	1/88	1.1%
Mallard	0/93	0.0%	0/18	U	11/72	15.3%
Egyptian goose	0/62	0.0%	1/28	3.6%	0/10	0%
Total	43/940	4.6%	12/347	3.5%	18/431	4.2%

U: unknown.

^a^ Data previously published [10].

^b^ Data (partly) previously published [10] and supplemented with Eurasian wigeon data from this study (n = 28) from 1 March 2016.

^c^ Data obtained in the current study from 23 October 2016 to 8 February 2017.

## Discussion

Here, we report on our virological findings in wild birds during the second wave of European HPAI H5(N8) outbreaks in 2016/17 and further investigate the use of serology in addition to virology in an outbreak situation. In this study we detected HPAI H5N8 viruses in 57 birds of 12 species. Initially, HPAI H5N8 virus was detected in dead wild birds by passive surveillance in mainly tufted ducks and Eurasian wigeons, followed by scavengers [16]. After these die-offs, the virus was detected in live wild birds and shifted from being found mostly Eurasian wigeons early in the outbreak towards mallards later in the outbreak, despite the fact that both species were screened throughout time. Although the number of HPAI H5(N8) infected wild birds identified by passive surveillance in this study and others [16-18] was much higher because of the massive die-offs and subsequent mandatory testing, the high virus prevalence in mallards would have been missed in passive surveillance studies since hardly any mallards were found dead and infected [16]. Likewise, the period of time of virus detection lasted longer in active surveillance compared with passive surveillance. Our results show that the mallard viruses from January 2017 were largely indistinguishable from the other HPAI H5N8 viruses, including those of tufted ducks, indicating that mallards might be more resistant to disease compared with other duck species, similarly to previous findings for HPAI H5N1 in mallards [26] and might therefore act as a reservoir species.

Results of analyses at the whole genome level indicated that the HA, NA and NS genes of Dutch H5N8 viruses were most closely related to 2014 HPAI H5N8 group B Eastern China viruses, while the other five genes were derived from Eurasian LPAI viruses. This genetic makeup is similar to viruses detected in Russia (May 2016) and Germany (autumn/winter 2016) [11,15]. Compared with the May 2016 Russian viruses, viruses in the Netherlands showed similar new reassortment events for the NP and PA genes as was described for the German viruses [15] (Table 5).

In contrast to the 2014/15 European emergence of HPAI H5N8, when a single lineage of HPAI spread across Europe, the chain of events during the 2016/17 HPAI H5 emergence shows more similarities to the 2014/15 situation in the United States (US), where the HPAI H5N8 group A viruses reassorted with local LPAI viruses causing massive and long lasting detection in both poultry and wild birds and local die-offs in wild birds [6]. While this manuscript was in preparation, detections of HPAI H5 clade 2.3.4.4 virus in Europe were still reported in Belgium, Luxembourg, the Netherlands and the United Kingdom [18], even though migrating birds had largely left their European wintering sites, suggesting that virus amplification was now occurring in local resident birds. This is a cause of concern, as establishment of HPAI viruses among wild birds is difficult to control and may give rise to a situation comparable to that in Asia with new outbreaks in wild birds and poultry not being caused by novel introductions of HPAI viruses from distant areas, but from within the local populations. It remains unclear, however, what drivers are responsible for the duration of virus circulation in a wild bird population, either long (US 2014/15 and Europe 2016/17) or short (Europe 2014/15), and based on current knowledge we cannot predict how the H5 situation among wild birds in Europe will evolve.

In case of introduction of new HPAI viruses, it would be highly beneficial to be able to target active surveillance to key species for virus detection to avoid excessive costs, sampling efforts, and inclusion of unnecessarily large numbers of animals. We therefore examined the use of experimental approaches for serology for the second time in an outbreak situation. To confirm serological data and to be able to determine the HPAI/LPAI and HPAI clade bias with some accuracy, we performed both HI and VN assays. Sera of 12 birds showed exclusive titres or a bias towards HPAI virus, seven to LPAI virus and seven remained ambiguous [24]. VN assays confirmed the presence of HPAI H5 clade 2.3.4.4-specific antibodies in 14/18 sera from October 2016 onwards and 1/2 from 1 March 2016. High cross-reactivity patterns and low initial titres in both assays showed that specifying biases towards one of the different HPAI H5 clades is very difficult. Further optimisation and validation of the assays are required to provide rough estimates of the seropositivity in subsequent years. Preliminary comparisons between the winters using the same HI assay starting from the 2014/15 winter show an outbreak-related incidence of HPAI H5 clade 2.3.4.4.-specific antibodies of 0% before 2014, rising to 4.6% during the first outbreak of HPAI H5N8 virus, decreasing to 3.5% in the 2015/16 winter and rising to 4.2% in the 2016/17 winter. Despite a similar antibody incidence between both outbreak periods, an apparent decreasing antibody incidence in two species detected throughout all three screening periods (Eurasian wigeons and mute swans) can be observed. This might be a consequence of differences in timing in peak prevalence between the first wave in 2014, with a very limited number of wild birds detected with a HPAI H5N8 infection (i.e. local virus amplification) [27,28] but high antibody incidence, and the current second wave, with substantial local virus replication and lower incidence of antibodies. These data could suggest that virus amplification in wigeons in 2014/15 took place before arrival of these birds in the Netherlands, whereas in 2016/17 virus amplification primarily took place within the Netherlands resulting in associated die-offs [14,16]. Unfortunately, we were unable to collect sera from wigeons late in the season in 2016/17 to confirm increasing antibody incidence. However, the mallards that were tested later in the outbreak (February 2017) showed an increase in antibody incidence after a peak in virus detections a few weeks earlier compared with those tested earlier in the outbreak (November–December 2016).

Recently, clade 2.3.4.4 H5N6 viruses started to circulate in both poultry and wild birds in south-east Asian counties [29] after their original detection in China [30], resembling HPAI H5N8 dispersion of 2014. In contrast to HPAI H5N8 viruses, these H5N6 viruses have caused sporadic human infections, including fatalities [31]. Hence it will be important to monitor the movements of these viruses by intense monitoring of wild bird populations in the coming winter seasons. In terms of multiple intracontinental spread of HPAI H5 viruses, global outbreaks were preceded by detections on breeding sites in Russia (Uvs-Nuur Lake district) and China (Qinghai Lake) after their initial detections in south-east Asia [11,32-35]. Increasing global collaborations and performing annual targeted active surveillance in China and on Russian breeding sites, and in Europe as autumn migration starts, will be important to provide early warning signals of HPAI virus dissemination.
